# [Retracted] Telemedicine in clinical setting (Review)

**DOI:** 10.3892/etm.2026.13118

**Published:** 2026-03-02

**Authors:** Xiao-Ying Zhang, Peiying Zhang

Exp Ther Med 12:2405–2407, 2016; DOI: 10.3892/etm.2016.3656

Following the publication of this article, an interested reader drew to our attention that the flow of the text, and the wording of numerous passages of text within the article, bore striking and unexpected similarities to the wording employed in a PhD thesis by Alexander Blum at Arizona State University in 2015 called “Evaluating Applications of a Telemedicine Taxonomy on the Classification of Research”.

After having conducted an internal investigation, the Editor of *Experimental and Therapeutic Medicine* has determined beyond all reasonable doubt that the review submitted to our Journal shared an unacceptable level of identity with the PhD thesis by Alexander Blum; therefore, this review article is to be retracted from the Journal. The Editor of *Experimental and Therapeutic Medicine* deeply regrets that this article was not intercepted by our own checks for plagiarism prior to the publication of the review, and sincerely apologizes to the author of the PhD thesis and the readership for the inconvenience caused. We also thank the interested reader for bringing this matter to our attention. The authors were asked for an explanation to account for these concerns, but the Editorial Office did not receive a reply.

